# Winemaking Technologies for the Production of Cabernet Sauvignon and Feteasca Neagra Wines Enriched with Antioxidant Active Principles Due to the Addition of Melatonin

**DOI:** 10.3390/foods13060884

**Published:** 2024-03-14

**Authors:** Sandra A. V. Eremia, Camelia Albu, Gabriel-Lucian Radu, Andreia Alecu, Alice G. Stoica, Elena Brinduse

**Affiliations:** 1 Centre of Bioanalysis, National Institute of Research and Development for Biological Sciences–Bucharest, 296 Splaiul Independentei, 060031 Bucharest, Romania; sandra.eremia@incdsb.ro (S.A.V.E.); lucian.radu@incdsb.ro (G.-L.R.); andreia.alecu@incdsb.ro (A.A.); alice.stoica@incdsb.ro (A.G.S.); 2 Institute for Research and Development for Viticulture and Wine Making, 2 Valea Mantei, Valea Calugareasca, 107620 Valea Calugareasca, Romania; elabrinduse@gmail.com

**Keywords:** winemaking process, Feteasca Neagra, polyphenols, antioxidant capacity, Cabernet Sauvignon, melatonin treatment

## Abstract

In recent years, various studies have been carried out to increase the concentration of antioxidant active principles in red wines as a consequence of the effects of winemaking techniques on the polyphenols content. In this study, in order to obtain the most optimal wine in terms of content and efficiency of antioxidant activity, various winemaking technologies (punching-down and pumping-over maceration) were tried with diverse gradations (Feteasca Neagra and Cabernet Sauvignon wines) and the addition of different concentrations of melatonin in must. Suitable HPLC and spectrophotometric methods were used to follow the evolution of the antioxidant compounds from wines during aging (for 12 months). After comparing the acquired results, an increase was observed in the antioxidant compound concentrations, particularly in resveratrol (85%), peonidin-3-glucoside (100%) or cyanidin-3-glucoside (100%), and antioxidant activity (10–40%). The most enriched wine was obtained in the case of Feteasca Neagra by the addition of 0.5 mg of melatonin per 1 kg of must using the punch-down technology and, in the case of Cabernet Sauvignon, by the addition of 0.05 mg of melatonin per 1 kg of must using the pumping-over technique. This study can provide winemakers with an approach to enhance red wines with antioxidant compounds.

## 1. Introduction

Wine is an economically relevant beverage that contains a variety of natural antioxidants and makes an important contribution to a healthy diet. The starting point for studies on red wine and health was the introduction of the term “French paradox”, which linked the low incidence of coronary heart disease to the population that has a moderate consumption of red wine [1]. Phenolic compounds are known to act as exogenous natural antioxidants [2], and research on wines has mainly focused on polyphenols [3], mainly flavonoids [4,5] and non-flavonoids [6,7]. An important antioxidant active principle contained, especially in red wines, is resveratrol, which reduces the risk of cardiovascular diseases and acts as a powerful antioxidant, both through a novel glutathione-sparing mechanism and through the classical uptake of hydroxyl radicals [8]. Red wines are the main source of resveratrol in the human diet.

Winemaking techniques are important for the concentration of phenolic compounds in red wines and can increase their extraction, particularly from the solid parts of the grapes (seeds and skins). The maceration conditions influence the phenolic composition and the biological activities of grape musts. For example, higher temperatures and longer maceration times result in a higher concentration of total anthocyanins [9]. Two classic methods of maceration–fermentation techniques are punching down and pumping over. During fermentation, a cap is formed from the solid components of the grapes, which is raised to the top of the fermentation tank via carbon dioxide production. To ensure contact between the juice and the skins, or seeds, they are usually mixed several times a day, either by pushing the cap under the juice (punching down) or by pumping the fermenting must out from the bottom of a tank and spraying it over the top of the tank (pumping over). Studies have been published comparing punching-down vinification with pumping-over. It was found that the extraction of polyphenolic compounds mainly depends on the grape varieties [10]. 

Feteasca Neagra (FN) is an old grape variety that mainly grows in Romania and the Republic of Moldova and from which a red wine with ruby-red reflections and an aroma of black raisins is produced, which becomes richer and softer with age. The FN wine is considered one of the best red wines from Romania and has a higher content of phenolic compounds than Pinot Noir [11]. Cabernet Sauvignon (CS) is the most widely cultivated grape variety in the world, producing a dark red wine with vanilla and dark fruit flavors. CS is considered the king of wines with a high tannin concentration and higher antioxidant activity compared to other red wines [12]. 

Some studies have been carried out to observe the effects of different winemaking techniques on the polyphenol content of CS. It was found that the vinification technique involving prolonged maceration (21 or 150 days) and implicitly the solid parts during the post-fermentative maceration phase significantly modulated the phenolic content and provided a higher content of polyphenolic compounds in steel tanks [13,14]. In wines with 16% saignée, a winemaking technique that involves the removal of a proportion of juice from a tank of crushed red grapes [15] increased the tannin content, and the results were long-lasting. Saignée also increased anthocyanin content in the first few days, but these results diminished by day 120 [16]. In young wines, the total anthocyanin content can be increased by the Ganimede fermenter, but the content decreases significantly after two years of aging, making the Ganimede fermenter suitable for the production of CS wines for early consumption [17]. It has been observed that the application of continuous treatment with pulsed electric fields resulted in obtaining a CS wine with a higher content of polyphenolic compounds and anthocyanins at the end of alcoholic fermentation [18]. In FN wines, it has been shown that the use of oak staves during vinification can lead to an increase in phenolic content, total antioxidant activity, the number of volatile compounds, and color intensification over time [19].

Many studies have demonstrated significant biological effects and evidenced that resveratrol from wines modulates various targets related to different biochemical pathways and can be considered as a therapeutic agent and as a protective agent against numerous diseases [20,21]. However, it is not possible to absorb the recommended therapeutic doses of resveratrol by drinking wine [22], and various studies have been focused on enhancing its concentration in wines. One factor that affects the presence of resveratrol in wine is the selected yeast strain used in alcoholic fermentation. To increase resveratrol content, yeast can be used with a higher ethanol-producing capacity, a pectolytic enzyme can be added, or transgenic microorganisms can also be added during maceration. Also, the extended maceration and malolactic fermentation could contribute to increased resveratrol levels in wine [23]. 

However, these approaches have various extraction yields and efficiency, and they may be feasible to increase the concentration of antioxidant active principle in red wine by increasing the maceration time and using the expensive tanks/oak in the winemaking process.

Melatonin, an important indoleamine compound with strong antioxidant activity [24], is present naturally in wines because it is extracted from grapes [25] and because it is biosynthesized from its precursors in some stages of the winemaking process [26,27]. Melatonin may have a synergistic effect with other antioxidants naturally present in wines [28] and may enhance the production of antioxidant compounds in the winemaking process. The exogenous administration of melatonin in must at the initial stage of the vinification process allows for the biosynthesis of polyphenolic compounds via phenylpropanoid metabolism and stimulates the activity of the enzymes phenylalanine ammonia lyase and cinnamic acid 4-hydroxylase to considerably modify the expression of specific genes for anthocyanin biosynthesis [29]. However, the research is still in an early stage, and it has not established what level of melatonin should be used and which winemaking process should be applied for the different grape varieties to improve red wines with antioxidant compounds. Currently, the addition of melatonin is not a legal practice foreseen on the list and description of the OIV Code of Oenological Practices referred to in the Commission Delegated Regulation [30]. However, the presence of melatonin in foodstuffs is allowed in certain European countries, and the European Food Safety Authority has issued a favorable opinion for two claims relating to the presence of melatonin in foodstuffs [31]. 

Through the recent detection of melatonin in beverages such as wine [32,33], an exciting new field of research has emerged, including the need for supplementary studies on melatonin-positive effects on polyphenolic profiles through its application in the winemaking process for quality improvements of wines with potential health benefits. 

This study aims to investigate different winemaking technologies with diverse gradations, starting with the addition of melatonin to the must to obtain a wine that is the most suitable in terms of content and efficiency of antioxidant activity. The aim of this study is to establish the most optimal winemaking technologies to produce red wines enhanced with antioxidant-active principles starting from the application of melatonin. Therefore, different melatonin concentrations, punching-down and pumping-over maceration techniques, and FN and CS grape varieties were evaluated to obtain the most efficient approach to improve wines with antioxidant activities. Appropriate chromatographic methods (HPLC) and spectrophotometry were used to monitor the evolution of the antioxidant compounds of the control and treated wines at the key aging stages of the wines (3, 6, 9, and 12 months).

## 2. Materials and Methods

### 2.1. Reagents

Melatonin (Sigma, Steinheim, Germany, M5250), chlorogenic acid (C3878), caffeic acid (C0625), (+)-catechin (43412), (−)-catechin (C0567), quercetin 3-glucoside (17793), quercitrin (Q3001), quercetin (Q4951), rutin (78095), resveratrol (R5010), cyanidin (79457), pelargonidin (PHL80084), Trolox (6-hidroxi-2,5,7,8-tetrametillchroman-2-carboxilic acid), chloroform (366919), ABTS (2,2-azinobis(3-ethylbenzothiaziline-6-sulfonate)), NaCl (S7653), sodium carbonate (Na_2_CO_3_), DPPH (2,2-diphenyl-1-picrylhydrazyl), potassium persulfate (K2S2O8), Folin & Ciocalteu’s phenol reagent (FCR), acetic acid (A6283), and formic acid (F0507) were bought from Sigma-Aldrich (Steinheim, Germany). Ellagic acid (45140), (−)-epicatechin (68097), gallic acid (48630), and myricetin (70050) were acquired from Fluka. Roth (Karlsruhe, Germany) has provided malvidin (6140.1), delphinidin (4537.1), petunidin-3-glucoside (89755), and peonidin-3-glucoside (1619.1) from PhytoLab (Dutendorfer, Germany). Delphinidin-3-glucoside, malvidin-3-glucoside and cyanidin-3-glucoside were procured from Polyphenols AS (Sandnes, Norway). All other used reagents, MeCN, methanol, and ethanol were chromatographic purity from Riedel-de Haen (Berlin, Germany). In total, 1 mg mL^−1^ of stock solutions were prepared in ethanol, taking into account the specific solubility of each standard and the chemical composition of the wines. 

### 2.2. Wine Sampling 

To accomplish the goal of this work, a 2 × 2 × 4 polyfactorial experiment was carried out with 16 variants in 4 replicates, in which the must was treated with melatonin in the preliminary stage. Three experimental factors were included with the following gradations:

- The variety of vines with gradations: FN and CS;
- The maceration–fermentation technique, with gradations: punching down (I) and pumping over (II);
- Melatonin concentration with gradations: 0 (M), 0.05 (V1), 0.1 (V2), and 0.5 (V3) mg of melatonin were applied per 1 kg of must.

FN and CS grape varieties from the geographical region of Valea Calugareasca, Romania, were used. Duplicate replications for each treatment/variant were carried out.

The grapes from FN and CS varieties were aleatorily harvested at technological ripeness (soluble sugar content according to the OIV-MA-AS2-02 Method was 210.1 ± 0.05 g L^−1^ for FN and 212.4 ± 0.14 g L^−1^ for CS; the total acidity according to the OIV-MA-AS313-01 Method [34] was 4.48 ± 0.12 g L^−1^ for FN and 4.10 ± 0.06 g L^−1^ for CS), destemmed, crushed and sulphurised (50 mg L^−1^ SO_2_). The wines were obtained for both grape varieties by the traditional method of maceration (duration of maceration in every treatment was 7 days), with half of the variants obtained by punching down, i.e., breaking the cap and immersing it in the must twice a day for 7 days, and the remaining samples by pumping over, i.e., extracting the juice from the bottom of the fermentation tank and pumping it over the cap once a day for 7 days. In the pre-vinification phase, 0.05, 0.1, and 0.5 mg of melatonin were applied per 1 kg of must. After decanting of experimental must, Actiflore F 33, *Saccharomyces cerevisiae* (20 g/hL) was added to start fermentation, and the wines were stirred in a stainless steel red wine tank to complete the fermentation and maturation process (12 months). Malolactic fermentation occurred spontaneously within 25–30 days. When the malolactic fermentation was complete and before bottling, the young wines were sulfited up to a level of 30 mg L^−1^ of free SO_2_. In this stage, the wines obtained from the FN variety were dry wines, with a content of residual sugar ranging between 2.7 and 4.0 g L^−1^, an alcoholic content of 12.5–13.1% vol., and a volatile acidity with values between 0.24 and 0.37 g L^−1^ (acetic acid). The wines obtained from the CS variety were dry wines, with a content of residual sugar ranging between 1.8 and 4.0 g L^−1^, an alcoholic content of 12.0–13.8% vol., and a volatile acidity with values between 0.20 and 0.42 g L^−1^ (acetic acid). The red wines produced were bottled after 12 months of aging. The storage conditions during the 12 months of the experiment were constant, and the wines were kept at a controlled cellar temperature between 10 and 15 °C.

For the monitoring of melatonin, a dispersive liquid–liquid microextraction with MeCN as the dispersing solvent and chloroform as the extraction solvent was used. A total of 8.5 mL of the centrifuged wine sample was transferred to a centrifuge tube and 1.5 mL of the chloroform/MeCN mixture at a ratio of 1:1, was added. After vortexing the samples for 10 min at 1200 rpm, 1 g of NaCl salt was added and then mixed again for 5 min. The next step was centrifugation at 5000 rpm for 5 min, after which the analyte of interest was found in the lower phase [35]. All wine samples were filtered into HPLC vials with a 0.2 µm Syringe Filter Unit (PTFE, Agilent, Beijing, China) and injected into the HPLC system.

To achieve the aim of this work and select the winemaking protocol of the most improved wine in terms of content and efficiency of antioxidant activity, monitoring of the active ingredients was carried out at the main aging stages of the wines: three, six, nine, and 12 months. 

### 2.3. HPLC Analysis

The HPLC analyses were accomplished using a Shimadzu complete system with two detectors, an RF-20A XS fluorescence detector and a mass spectrometer detector, LCMS-2010. For melatonin analysis, the fluorescence detector parameters were as follows: λ_ex_ = 285 nm and λ_em_ = 340 nm; cell temperature control at 250 °C; 1.5 s response; Gain × 4; medium sensitivity. For polyphenol compounds analysis, the MS parameters were as follows: ESI interface, CDL temperature, 200 °C; heat block temperature, 200 °C; nebulization gas (N_2_) flow rate, 1.5 L min^−1^; detector voltage, 1.8 kV, interface voltage 4 kV and interface temperature, 250 °C. 

The identification and quantification of melatonin by HPLC-FL were performed using a previously developed method [35], a Kromasil 100-5-C18 2.1 × 100 mm column, an elution gradient of mobile phase (methanol, solvent A, and 1% acetic acid, solvent B) and flow rate of 1 mL min^−1^. The analysis time was 30 min and the column temperature was 20 °C.

The analysis of polyphenolic compounds was achieved on a C18 column, Kromasil, 100-3.5, 2.1 × 100 mm, with a mobile phase, pH = 3, composed from water with formic acid, and MeCN with formic acid. An elution and a flow rate gradient of the mobile phase was used [11].

The MS detection was performed using negative ionization mode and the selected ion monitoring (SIM) mode was used to obtain the corresponding peaks of the polyphenolic compounds fragment ions ([M − H]: 169, 179, 227, 289, 301, 317 353, 447, 463 and 609).

The anthocyanidins and anthocyanins quantification was achieved through a C18 Kromasil column, 100-3.5 4.6 × 50 mm, with a gradient of mobile phase (5% formic acid in water and 5% formic acid in methanol), a flow rate of 0.2 mL min^−1^, and the temperature column at 40 °C [36]. The positive ionization mode was used to obtain the following corresponding peaks of the compound fragment ions ([M − H]^+^: 493, 479, 465, 463, 449, 331, and 303) in SIM mode.

### 2.4. Trolox Equivalent Antioxidant Capacity (TEAC) Assay Using ABTS·+

ABTS·+ is a radical cation formed by the oxidation of ABTS (7 × 10^−3^ M) with K_2_S_2_O_8_ (2.5 × 10^−3^ M). For the stabilization of ABTS·+, the solution was kept for at least 20 h at room temperature in the dark. The solution was obtained by diluting (1:40 *v/v)* the stock solution and combining it with water and sampled at a ratio of 25:4:1, ABTS·+/solvent:/sample, *v/v/v*. The absorbance was measured at 735 nm with a Thermo Spectrophotometer (Thermo Fischer Scientific, Waltham, MA, USA). The obtained values were presented in relation to a compound considered a reference antioxidant, Trolox equivalent (8.5 × 10^−6^ M) [37].

### 2.5. TEAC Assay Using DPPH•

The stable radical, DPPH• (2.5 × 10^−4^ M), was formed by dissolving DPPH in ethanol. The solution was mixed with ethanol and sampled at a ratio of 1:1.9:0.1, DPPH•/solvent/sample, *v/v/v*. The decrease in DPPH was measured at 515 nm. The results were expressed as Trolox (8.5 × 10^−6^ M) equivalent [38].

### 2.6. Folin & Ciocalteu Assay

The FCR was diluted in ultrapure water (1:10) and mixed at a ratio of 5:1, *v/v,* with the phenol compound. After 10 min, to obtain a blue complex between the polyphenol compounds and the Folin and Ciocalteu, 7.8% Na_2_CO_3_ was pipetted in the alkaline medium [39]. After 1 h, the complex absorbance was measured at 766 nm. The results attained were presented in relation to a reference polyphenol in wines, namely gallic acid. 

Each sample of standards and wines was analyzed in triplicate. The results were presented using descriptive statistics, which summarizes data using indexes such as mean, median, and standard deviation (SD), as mean ± SD. Also, the data were analyzed by a one-way analysis of variance (ANOVA) using Excel software, 2021. ANOVA was used to determine the significant differences, the probability (*p* < 0.05), and the variability (F and F-critical values) between the means.

## 3. Results and Discussion

For the monitoring of active principles from red wines, the performance characteristics of the HPLC methods required for the analytical analyses were first determined. From chromatograms obtained, a clear difference was observed between the specific peaks, and the retention times were between 3.27 and 35.83 min for polyphenolic compounds, between 23.98 and 35.25 min for anthocyanidins/anthocyanins, and 23.58 min for melatonin. The peaks were properly resolved with a suitable resolution in the case of catechin compounds ([M − H] = 289) and quercetin and ellagic acid ([M − H] = 301). The values obtained for the range of response (0.5–50 µg mL^−1^ for polyphenolic compounds and 1–30 ng mL^−1^ for melatonin analysis), the correlation coefficients (R) between 0.9991 and 0.9999, the limit of detection (LoD), which was less than 0.44 µg mL^−1^ for polyphenolic compounds and 0.01 ng mL^−1^ for melatonin analysis, and the limit of quantification (LoQ) (0.05 ng mL^−1^ for melatonin analysis and less than 0.48 µg mL^−1^) prove that these three methods are appropriate for the analysis of polyphenols compounds, anthocyanidins/anthocyanins, and melatonin from the 16 wine samples. 

### 3.1. The Evolution of Melatonin Contents in Profiles in the Aging Process of FN and CS Wines

The HPLC-FL analysis of the wine samples provides important information about the melatonin reference values in the grape variety and, in our case, in the 16 variants of the wine samples. Based on this information, we can determine the most promising melatonin concentration to be applied in the must and identify which grape varieties lead to the highest melatonin content obtained in winemaking. These results also help to understand the evolution of the polyphenol compounds or antioxidant activity during the aging process of red wines. The results from HPLC-FL analyses are presented in Table 1.

Based on the results obtained after 3 and 6 months, the biosynthesis of melatonin was observed (except for variant 3, FNIV3, FNIIV3, and CSIV3, where the addition of melatonin was 0.5 mg per 1 kg of must and biosynthesis at the level of ng mL^−1^ was not observed), confirming the fact that the low temperatures made the fermentation extremely slow, requiring a longer period in the winemaking process. During fermentation, melatonin is synthesized by the yeasts from L-tryptophan and serotonin [40]. At this stage, L-tryptophan is released into the wine, and serotonin is obtained from the decarboxylation of L-tryptophan by the action of yeast, lactic acid bacteria, or other contaminating microorganisms [41]. The final melatonin concentration can be influenced by the fermentation conditions, the L-tryptophan concentration, and the sugar content [42]. A total of 9 months after the start of vinification, a decrease in melatonin concentration of about 30% was observed in the wines, which correlates with the positive trend observed between 6 and 9 months for the concentrations of different polyphenolic compounds. After 12 months, the melatonin concentrations observed show a slight downward trend, with values comparable to those observed after 9 months, which indicates the end of the wine’s aging process and the slowing down of biochemical and chemical reactions.

### 3.2. The Evolution of Phenolic Acids, Flavanols, Flavonols, and Stilbenes Profiles in the Aging Process of FN and CS Wines

Three months after the start of the vinification, monitoring of the polyphenolic profiles of the FN wines revealed that the concentrations of polyphenolic compounds in the melatonin-treated wines were similar to those in the control wines. Higher concentrations were obtained for the wine treated with 0.5 mg of melatonin per 1 kg of must, with the best results obtained using the punching down technique (FNIV3).

In the 6-month phase, the previous results were maintained with FNIV3 as the most improved wine. It was found that in a young wine, when the concentration of catechins, caffeic acid, or chlorogenic acid from FN is sought, the pumping-over technique can be used, and when the concentration of quercetin compounds is desired, the punching down technique is recommended. Nine months after the start of vinification, the results showed that the concentration values for 11 polyphenolic compounds, except gallic acid, were higher in all treated FN wines than in the control wines, indicating that the punching-down technique is the most suitable to obtain better wines. Quantitatively, the monitored compounds were 50% higher than those of the control sample when 0.5 mg of melatonin as applied per 1 kg of must. After 12 months, the concentrations of polyphenolic compounds began to decrease in all wines and the FNIV3 was the most improved wine.

In the case of CS wines, slight improvements in the concentrations of polyphenolic compounds were observed after 3 months in the treated wines in comparison to the control wines. Even if the differences are small between the extraction yield of polyphenolic compounds when punching down is used and the one obtained using pumping over, which depends on different variables, it was shown that the pumping-over technique gave all varieties of wines significantly higher quercetin levels [43]. CS compared to FN has a higher content of quercetin compounds, which makes pumping over more suitable for obtaining wines with increased antioxidant compounds for both the control wine and the variants. The highest concentrations for these wines were obtained for the wine treated with 0.05 mg of melatonin per 1 Kg of must when the pumping-over technique was used (CSIIV1). The results obtained after 6 months remained constant, confirming that pumping over is the most optimal technique in the case of CS wine. 

Based on 9 months of data, it was found that the maturation process in all CS wines continued up to 12 months, the reactions were still implicated in the biosynthesis of the monitored polyphenols compounds, and the CSIIV1 wine improved by 15% through melatonin treatment. After 12 months, the concentrations of polyphenolic compounds were generally highest in all wines, except for quercetin compounds where a decrease was observed and rutin was not identified, and better results were obtained in the treated wines than in the control wines. In the CSIIV1 wine, the quantitative values of the monitored compounds increased by about 25%.

**Figure 1:**
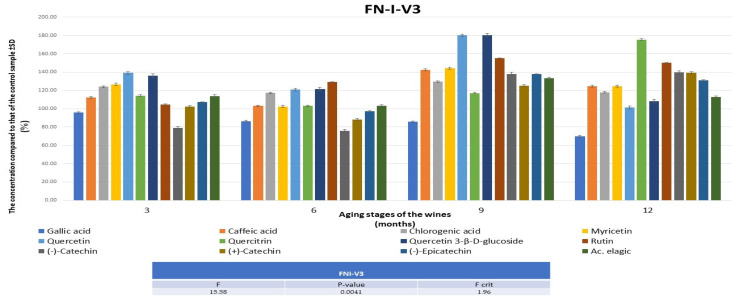
The evolution of the concentrations of polyphenols compounds in FNIV3 compared to the data obtained for the control wine during 12 months. FN—Feteasca Neagra; I—punching down; V3—0.5 mg of melatonin was applied to 1 kg of must.

**Figure 2:**
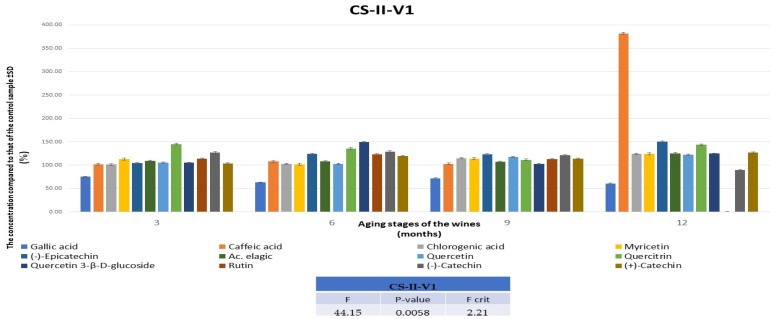
The evolution of the concentrations of polyphenols compounds in CSIIV1 compared to the data obtained for the control wine during 12 months. CS—Cabernet Sauvignon; II—pumping over; V1—0.05 mg of melatonin were applied to 1 kg of must.

The results obtained after 3 months show that the most improved wines were FNIV3 and CSIIV1 and that the most optimal techniques were punching down for FN and pumping over for CS. Figure 1 and Figure 2 show the polyphenolic profile evolution of these red wines over time (3, 6, 9, and 12 months) compared to the control wines.

In these wines, the analytes of interest were quantified at higher concentrations than in the control samples, except for gallic acid in both FNIV3 and CSIIV1 and catechin, especially in FNIV3. An increase was observed in gallic acid during the aging of the wines, and a lower amount was observed in the treated wines, but this is in agreement with the results obtained for the monitoring of anthocyanidins and anthocyanins, considering the shikimate/phenylpropanoid metabolism. It is possible that, in the presence of melatonin, the conversion into ellagic acid of gallic acid increased.

Regarding the evolution of resveratrol concentration from the 16 varieties of the wines monitored, the results confirmed that the addition of melatonin leads to an increase in the concentration of this principle active in red wines. In FN wines, the highest concentration of resveratrol was obtained at 3 months after the start of the vinification process (9.57 ± 0.02–19.28 ± 0.21 µg mL^−1^), followed by a linear decrease in the next 6 months and an improvement after 12 months of wine aging (7.98 ± 0.08–14.81 ± 0.12 µg mL^−1^). In CS wines, the content of resveratrol increased during the aging of the wines, both in the control and in the treated wines, and at 12 months, the best results were obtained with values of 11.83 ± 0.14–21.40 ± 0.21 µg mL^−1^. The best improvements in terms of resveratrol content in wines were obtained for FNIV3 and CSIIV1, respectively (Table 2). In the case of FNIV3, a 50% increase (19.28 ± 0.21 vs. 12.85 ± 0.18 µg mL^−1^) was achieved in young wines (3 months), and after 12 months, an 85% intensification in the resveratrol content in the treated wine (14.81 ± 0.24 µg mL^−1^) was observed compared to the control wine (7.98 ± 0.03 µg mL^−1^). In the case of CSIIV1, approximately 45% increase was observed in all the monitored significant steps of the vinification process, and after 12 months, a 54% intensification was obtained in the resveratrol content in the treated wine (21.4 ± 0.28 µg mL^−1^) compared to the control wine (13.9 ± 0.21 µg mL^−1^). The treatment with melatonin improved the content of resveratrol both in FN wines and in CS wines, proving that a better winemaking technology for CS resulted in increased resveratrol compared to the thermovinification and separation of must from pomace [44]. The results attained are comparable to those previously published after studying the effect of 70 different strains of Saccharomyces cerevisiae on the content of resveratrol in CS wine [45].

### 3.3. The Evolution of Anthocyanidins/Anthocyanins Profiles in the Aging Process of FN and CS Wines

**Figure 3:**
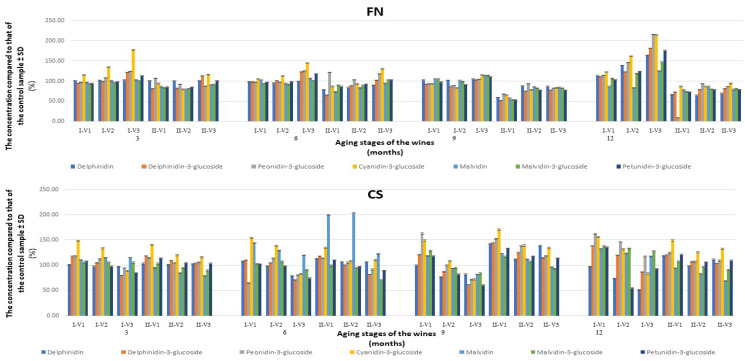
The concentration values of the 7 anthocyanidins/anthocyanins obtained for the 12 varieties of treated wines compared to the data obtained for the control wines, during the aging process (3, 6, 9, and 12 months, *p*-value = 0.0098 for FN, respectively, *p*-value = 0.0038 for CS). FN—Feteasca Neagra, CS—Cabernet Sauvignon, I—punching down, II—pumping over, V1—0.05, V2—0.1, and V3—0.5 mg of melatonin were applied to 1 kg of must.

From the results obtained following the monitoring of seven anthocyanidins/anthocyanins in the key points of the wine maturation process, it appears that in the case of the treated FN wines, to obtain wines with higher concentrations of anthocyanins, the recommended technique is punching down (Figure 3). The wines obtained by pumping over generally have lower concentrations of the analytes of interest than the control wines. An explanation of this would be the fact that anthocyanin biosynthesis appears to be in competition with kaempferol 3-*O*-glycosides production usually glycosylated by the catalysis of UDP-D-glucose flavonoid 3-*O*-glycosyltransferase [46]. The treatment with melatonin in red wines leads to an increase in the enzyme activity of UDP-D-glucose flavonoid 3-*O*-glycosyltransferase, and it is possible that kaempferol 3-*O*-glycosides synthesis may be intensified in treated wines to the detriment of anthocyanins. The only promising wine is the one treated with 0.5 mg of melatonin per 1 kg of must, but only if it is in the first months of aging (3 or 6 months).

**Figure 4:**
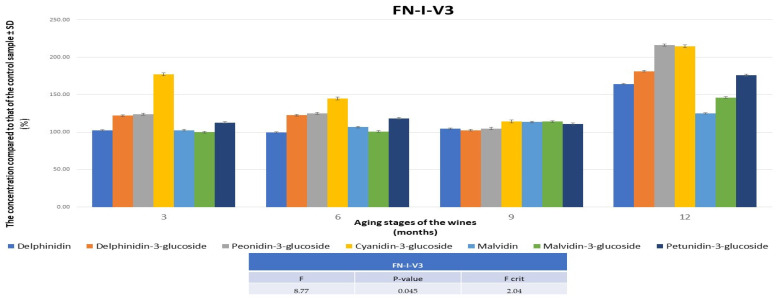
The evolution of the concentrations of anthocyanidins/anthocyanins in FNIV3, in comparison with the data obtained for the control wine during 12 months. FN—Feteasca Neagra; I—punching down; V3—0.5 mg of melatonin was applied to 1 kg of must.

The V3 variant differs from the wines obtained using the punching down technique, especially after 12 months, when a quantitative improvement in anthocyanins of over 60% was achieved compared to the concentrations in the control wine (Figure 4). These results are in line with those observed in the monitoring of polyphenolic compounds.

**Figure 5:**
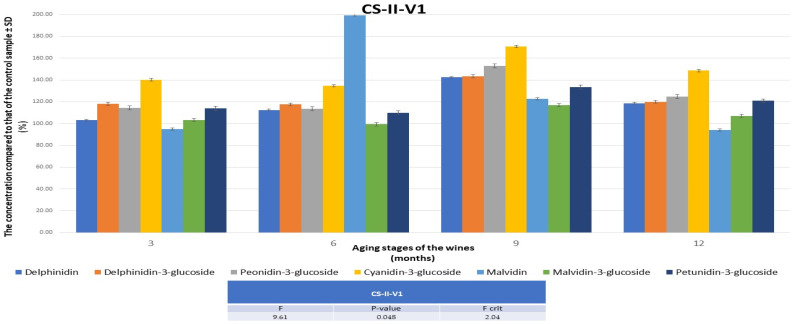
The evolution of the concentrations of anthocyanidins/anthocyanins in CSIIV1, in comparison with the data obtained for the control wine during 12 months. CS—Cabernet Sauvignon; II—pumping over; V1—0.05 mg of melatonin was applied to 1 kg of must.

Regarding the CS wines, following HPLC-MS analyses for the quantification of anthocyanidins/anthocyanins for the 6 wine varieties, it was found that both maceration–fermentation techniques led to improved wines, and the lowest values were observed after the addition of 0.5 mg of melatonin per 1 kg of must. The best results were consistently achieved with the addition of only 0.05 mg of melatonin per 1 kg of must, with wines being enhanced by at least 12% after 3 months, reaching 40% after 9 months, and then reducing to 25% at 12 months. Comparing the two V1variants, and considering that at 3, 6, and 9 months, respectively, better results were obtained for the wine produced with the pumping-over technique, the CS-II-V1 variant was selected as the optimal improved wine. The evolution of anthocyanidins/anthocyanins for this wine variant over 12 months is presented in Figure 5. The results obtained were in accordance with those obtained from the HPLC-MS analyses of the polyphenol compounds (Table S1). The results prove that the addition of melatonin has effects comparable to those obtained by the addition of caffeic and rosmarinic acids before alcoholic fermentation in terms of the anthocyanin content of CS wines [47].

### 3.4. The Evolution of Antioxidant Activity in the Aging Process of FN and CS Wines

When studying the antioxidant active principles, it is advisable to conduct qualitative/quantitative HPLC-MS analyses with the total phenolic content (TPC) assay and to use more than one method for antioxidant activity. In this study, the DPPH and ABTS methods, which hinge on electron transfer and imply the reduction in an oxidizing agent, were selected to estimate the antioxidant activity of red wines and to correlate these assays. 

To evaluate the antioxidant activity of FN and CS wines, operational parameters such as the dilution factor used depending on the sample content, the specific measurement wavelength, and the maximum absorbance value were first determined. To determine the total content of polyphenols in the sample, the calibration curve of the reference polyphenol gallic acid was also established for seven different concentrations in the range of 0.01–0.1 mg mL^−1^. 

**Figure 6:**
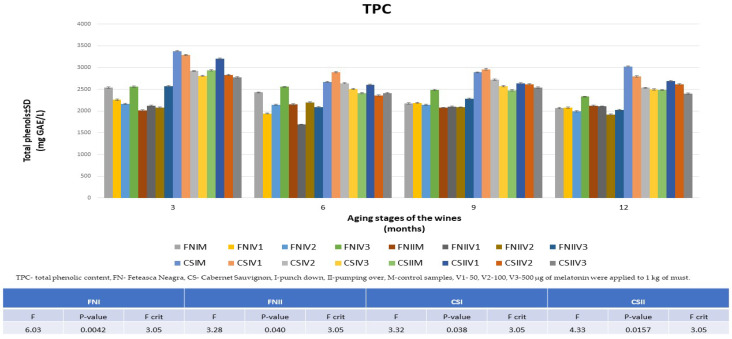
Comparison of the total content of polyphenols in the 12 varieties of treated wines compared to the data obtained for the control wines during the aging process (3, 6, 9, and 12 months and ANOVA results).

Based on the data values obtained from the TPC analysis over 12 months (Figure 6), the results were found to be consistent with those of the HPLC analyses for the polyphenolic compounds. The gallic acid equivalent (GAE) values found were in the range of 1687 ± 22–2574 ± 31 GAE mg L^−1^ for the FN wines and 2353 ± 29–3378 ± 35 GAE mg L^−1^ in the case of the CS wines. Similar to the HPLC analysis, the most improved wines with melatonin treatment were FNIV3 and CSIIV1. After 12 months from the start of vinification, the values in these wines were 14% higher for FNIV3 and 9% higher for CSIIV1 compared to the control wines.

**Figure 7:**
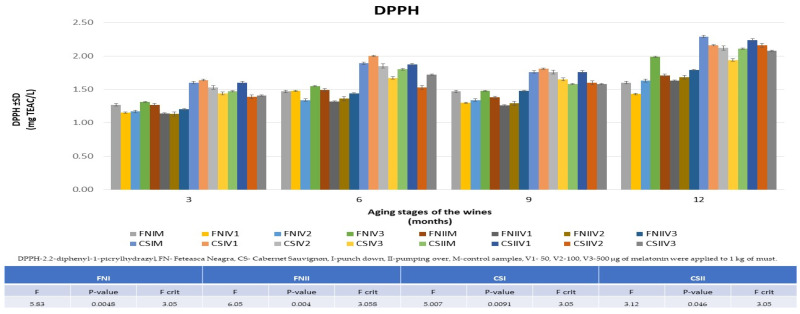
Comparison of the results obtained from the DPPH assay for the 12 varieties of treated wines compared to the data obtained for the control wines during the aging process (3, 6, 9, and 12 months and ANOVA results).

Figure 7 shows that one of the effects of treating the wines with melatonin is to increase the capability to scavenge DPPH free radicals, with the highest increases in FNIV3 at 24% and CSIIV1 at 11%. Furthermore, it is observed that the data values obtained after 12 months for CS wines (CSIIM: 2.02 ± 0.05 TEAC mg L^−1^ and CSIIV1: 2.25 ± 0.05 TEAC mg L^−1^, respectively) are higher than those for FN wines (FNIM: 1.6 ± 0.01 TEAC mg L^−1^ and FNIV3: 1.99 ± 0.02 TEAC mg L^−1^, respectively), and the results are consistent with those obtained for TPC.

**Figure 8:**
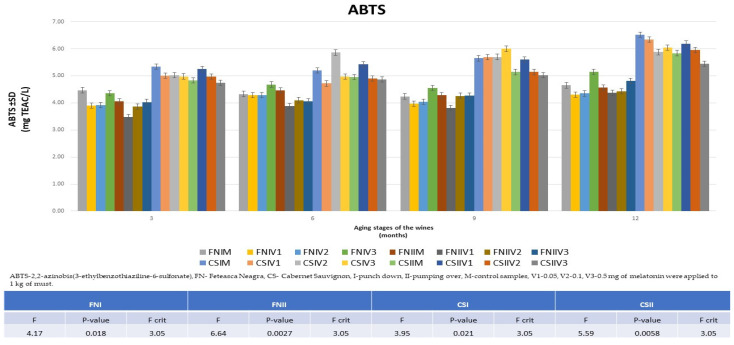
Comparison of the results obtained from ABTS assay for the 12 varieties of treated wines compared to the data obtained for the control wines during the aging process (3, 6, 9, and 12 months and ANOVA results).

The results obtained for the ABTS·+ radical scavenging capacity in the FN and CS wine samples did not differ significantly, and an intensification of the antioxidant activity by treating the wines with melatonin was obtained for the FNIV3 wine after 12 months and for the CSIIV1 wine after 9 months (Figure 8). Also, in this case, higher values were found for the CS wines (4.72 ± 0.05–6.52 ± 0.04 TEAC mg L^−1^) compared to the FN wines (3.48 ± 0.02–5.15 ± 0.04 TEAC mg L^−1^). The differences correlate with the data obtained for TPC and DPPH.

The intensification in the antioxidant activity obtained by treatment with melatonin (~10%), reflected in the DPPH and ABTS results, is not significantly different from that obtained in CS wines that were produced by using a mixed fermentation of *Pichia *kudriavzevii** M759 and *Saccharomyces cerevisiae* 7VA [48]. 

After the quantitative monitoring of polyphenolic compounds, based on anthocyanidins/anthocyanins, total polyphenol content, and antioxidant activity of the 12 red wines produced by the addition of melatonin in the preliminary phase of vinification, it can be concluded that in the case of FN wines, antioxidant activity can be significantly increased by the addition of 0.5 mg of melatonin per 1 Kg of must and the application of the punch-down maceration method. In the case of CS wines, remarkable increases are achieved by adding 0.05 mg of melatonin per 1 Kg of must using both maceration and fermentation techniques, with the antioxidant-active ingredients being retained for 12 months by using the pumping-over technique.

## 4. Conclusions

In this work, after studying diverse technologies with different gradations, it can be concluded that the most suitable wine in terms of content and efficiency of antioxidant activity can be obtained, in the case of FN, by the addition of 0.5 mg of melatonin per 1 kg of must and the application of the punch-down maceration technology, and in the case of CS, by the addition of 0.05 mg of melatonin per 1 kg of must using the pumping-over technique. The addition of melatonin in the initial period of vinification process significantly increases the concentrations of resveratrol (85%), peonidin-3-glucoside (over 100%), or cyanidin-3-glucoside (over 100%) in FN (12 months), and resveratrol (54%) or cyanidin-3-glucoside (50%) in CS (12 months) compared with those from control wines. Also, regarding antioxidant activity, reflected in the DPPH and ABTS results, FN wine improved by at least 40% after 9 months and CS wine improved by ~10% compared to control wines. 

The data obtained for Cabernet Sauvignon is comparable to data previously reported in the literature and support that the studied technology is an optimal tool to obtain a wine improved in terms of content and efficiency of antioxidant activity. However, further studies are necessary to monitor the repeatability and the reproducibility of antioxidant activity and the evolution of the profile of polyphenol compounds during the aging process of these two red wines produced using the proposed optimal technologies.

## Figures and Tables

**Table 1:** The melatonin values obtained by the HPLC-FL method from 16 variant wine samples (mean ± standard deviation and ANOVA results).

Melatonin ng mL^−1^							
FNIM	FNIV1	FNIV2	FNIV3	FNIIM	FNIIV1	FNIIV2	FNIIV3
3 months							
0.74 ± 0.01	12.99 ± 0.09	30.31 ± 0.12	193.65 ± 1.11	1.09 ± 0.02	20.50 ± 0.14	30.22 ± 0.17	158.17 ± 1.58
6 months							
1.92 ± 0.02	14.90 ± 0.14	31.41 ± 0.21	192.33 ± 1.57	2.10 ± 0.01	24.49 ± 0.15	35.43 ± 0.24	154.33 ± 1.85
9 months							
1.80 ± 0.03	9. 67 ± 0.10	20.18 ± 0.15	124.69 ± 1.91	3.13 ± 0.03	19.28 ± 0.17	27.24 ± 0.23	107.55 ± 1.62
12 months							
1.63 ± 0.02	9. 18 ± 0.11	20.08 ± 0.17	111.1 ± 1.31	1.14 ± 0.02	18.59 ± 0.13	26.88 ± 0.31	102.76 ± 1.28
F	*p*-value		F crit	F	*p*-value		F crit
42.31	1.17 × 10^−6^		3.49	59.373	1.8 × 10^−7^		3.49
CSIM	CSIV1	CSIV2	CSIV3	CSIIM	CSIIV1	CSIIV2	CSIIV3
3 months							
0.84 ± 0.02	16.19 ± 0.09	39.28 ± 0.11	158.71 ± 1.26	1.36 ± 0.04	24.41 ± 0.22	40.16 ± 0.31	165.34 ± 1.72
6 months							
2.53 ± 0.02	27.89 ± 0.41	43.63 ± 0.38	154.99 ± 1.57	1.43 ± 0.01	25.67 ± 0.24	47.05 ± 0.38	177.47 ± 1.57
9 months							
2.06 ± 0.5	11.28 ± 0.28	30.67 ± 0.27	114.64 ± 1.37	2.96 ± 0.02	17.12 ± 0.23	26.87 ± 0.15	102.39 ± 0.95
12 months							
0.31 ± 0.01	6.65 ± 0.22	25.46 ± 0.23	89.66 ± 0.95	1.40 ± 0.02	16.93 ± 0.13	25.99 ± 0.22	92.31 ± 0.82
F	*p*-value		F crit	F	*p*-value		F crit
42.53	1.14 × 10^−6^		3.49	28.00	1.06 × 10^−5^		3.49

FN—Feteasca Neagra, CS—Cabernet Sauvignon, I—punching down, II—pumping over, M—control samples; V1—0.05, V2—0.1, and V3—0.5 mg of melatonin were applied to 1 kg of must.

**Table 2:** The evolution of the resveratrol concentrations in FNIV3, respectively, and CSIIV1, in comparison with the data obtained for the control wines during 12 months (mean ± standard deviation and ANOVA results).

Resveratrol µg mL^−1^			
FNIM	FNIV3	CSIIM	CSIIV1
3 months			
12.85 ± 0.18	19.28 ± 0.21	9.29 ± 0.06	13.2 ± 0.12
6 months			
9.33 ± 0.05	11.5 ± 0.08	10.52 ± 0.07	15.73 ± 0.15
9 months			
7.58 ± 0.08	10.33 ± 0.09	13.68 ± 0.11	19.13 ± 0.14
12 months			
7.98 ± 0.03	14.81 ± 0.24	13.9 ± 0.21	21.4 ± 0.28
F	*p*-value		F crit
4.49	0.24		3.49

FN—Feteasca Neagra, CS—Cabernet Sauvignon, I—punching down, II—pumping over, and M—control samples; V1—0.05 and V3—0.5 mg of melatonin were applied to 1 kg of must.

## Data Availability

The original contributions presented in the study are included in the article/Supplementary Materials, further inquiries can be directed to the corresponding author.

## Supplementary Materials

The following supporting information can be downloaded at: https://www.mdpi.com/article/10.3390/foods13060884/s1, Table S1: The values obtained by the HPLC-MS methods from red wines I add citation in main.
