# Supplementary material for: Winemaking Technologies for the Production of Cabernet Sauvignon and Feteasca Neagra Wines Enriched with Antioxidant Active Principles Due to the Addition of Melatonin
Source: Foods. 2024 Mar 14;13(6):884. doi: 10.3390/foods13060884 (PMC10969225; doi:10.3390/foods13060884)
Supplement: Supplementary file 1 [file foods-13-00884-s001.zip › foods-2901310-supplementary.pdf]

Table S1. The phenolic acids, flavanols, flavonols and stilbenes values obtained by the HPLC-MS method from red wines

µg mL^{-1}

Compound	FNI M	FNI V1	FNI V2	FNI V3	FNII M	FNII V1	FNII V2	FNII V3	CSI M	CSI V1	CSI V2	CSI V3	CSII M	CSII V1	CSII V2	CSII V3
Gallic acid	3 months (december 2022)															
	16.32	13.72	14.27	15.62	20.76	17.81	14.76	14.48	13.60	9.91	9.76	11.80	12.14	9.13	9.49	7.94
	6 months (march2023)															
	150.68	130.98	129.07	130.09	153.17	137.43	132.84	123.37	119.78	88.17	74.12	77.10	70.29	44.38	50.00	61.09
	9 months (june 2023)															
	192.16	187.24	187.46	164.72	187.63	164.37	141.16	151.89	159.67	123.47	110.04	148.79	117.19	84.19	71.61	64.57
	12 months (september 2023)															
	252.50	204.59	205.68	176.46	186.61	149.90	119.31	96.05	393.47	186.47	215.20	194.93	373.90	227.91	213.28	169.76
Caffeic acid	3 months (december 2022)															
	9.79	9.02	8.83	10.98	11.22	14.02	12.47	12.26	3.79	3.71	3.59	3.36	4.63	4.73	4.59	3.59
	6 months (march2023)															
	5.29	4.98	4.63	5.45	5.19	6.00	6.37	5.34	1.89	1.97	1.59	1.36	1.62	1.75	1.57	1.02
	9 months (june 2023)															
	11.25	13.37	12.62	16.04	15.30	19.28	16.33	16.48	7.06	6.36	5.35	6.58	5.37	5.53	4.19	3.32
	12 months (september 2023)															
	5.33	6.44	5.16	6.65	3.79	3.30	3.03	1.84	3.87	4.67	5.38	7.17	1.61	6.15	5.11	3.41
Chlorogenic acid	3 months (december 2022)															
	6.25	6.52	6.84	7.75	7.21	8.28	6.89	6.46	10.75	12.93	11.26	12.07	11.11	11.27	11.12	9.82
	6 months (march2023)															
	5.48	5.85	5.72	6.42	3.80	4.08	4.23	5.38	7.70	9.37	8.21	7.52	6.73	6.93	6.50	6.58
	9 months (june 2023)															
	3.60	4.40	3.88	4.66	4.84	5.29	4.71	5.61	5.68	5.30	5.10	4.92	3.73	4.30	3.89	2.95
	12 months (september 2023)															
	3.91	3.63	4.48	4.61	3.52	3.06	2.90	2.33	5.53	6.71	5.74	10.27	8.30	10.31	8.32	5.46
Myricetin	3 months (december 2022)															
	25.47	22.68	24.80	32.26	31.24	22.66	18.39	28.76	21.71	22.30	23.80	21.91	26.75	30.20	28.53	21.67
	6 months (march2023)															
	22.07	19.85	18.73	22.62	20.19	15.41	16.28	18.81	13.76	14.69	14.94	11.53	13.48	13.71	12.26	10.72
	9 months (june 2023)															
	28.69	30.74	29.28	41.38	43.33	33.17	30.80	43.19	26.81	28.10	27.34	22.94	25.98	29.62	24.29	18.82
	12 months (september 2023)															
	19.73	21.55	16.93	24.57	13.82	7.39	7.76	6.70	9.68	10.83	18.58	24.84	24.70	30.74	21.39	14.41
Quercetin	3 months (december 2022)															
	16.59	17.72	19.17	23.07	24.49	17.66	13.62	20.20	27.06	26.09	27.09	23.78	28.91	30.41	29.10	25.92

	6 months (march2023)															
	17.37	15.21	14.50	20.98	19.70	12.05	13.08	12.11	23.19	22.00	22.46	17.39	18.08	18.55	17.54	15.40
	9 months (june 2023)															
	13.10	13.52	10.97	23.59	28.25	16.34	16.59	15.37	30.08	28.86	27.90	20.41	23.42	27.52	22.36	18.74
	12 months (september 2023)															
	9.26	8.13	5.24	9.39	7.25	3.37	3.62	2.26	15.14	15.21	22.99	31.07	26.58	32.58	24.05	19.26
Quercitrin	3 months (december 2022)															
	0.14	0.14	0.16	0.16	0.16	0.12	0.15	0.15	4.48	3.88	2.78	2.24	2.09	3.03	2.03	2.44
	6 months (march2023)															
	0.33	0.34	0.35	0.34	0.34	0.30	0.32	0.32	4.15	3.61	2.84	1.86	1.82	2.47	1.64	1.93
	9 months (june 2023)															
	0.94	0.99	0.97	1.10	0.92	0.95	0.88	0.86	7.74	7.06	5.23	3.49	3.37	3.76	2.94	3.57
	12 months (september 2023)															
	0.53	0.76	0.65	0.93	0.47	0.33	0.37	0.33	3.05	2.84	2.73	2.13	1.90	2.73	2.09	2.25
Quercetin 3-β-D-glucoside	3 months (december 2022)															
	3.78	4.00	4.05	5.14	5.46	2.93	3.07	3.07	46.27	42.43	39.18	34.18	35.67	37.64	33.48	38.96
	6 months (march2023)															
	3.37	3.80	3.61	4.09	4.01	2.15	2.33	2.59	20.33	15.74	14.33	11.09	10.86	16.27	10.90	10.31
	9 months (june 2023)															
	5.13	8.03	7.61	9.25	8.39	5.39	4.66	5.64	109.39	100.17	90.76	73.40	71.49	73.72	59.99	66.49
	12 months (september 2023)															
	1.48	1.62	1.35	1.60	1.07	0.56	0.51	0.43	26.55	27.20	25.37	22.81	23.69	29.65	21.36	21.90
Rutin	3 months (december 2022)															
	1.30	1.16	1.10	1.36	1.49	1.07	1.08	1.27	4.74	4.88	3.66	2.59	2.86	3.26	2.81	3.05
	6 months (march2023)															
	0.62	0.62	0.62	0.80	0.85	0.70	0.73	0.73	2.76	2.71	2.12	1.22	1.29	1.59	1.22	1.48
	9 months (june 2023)															
	1.60	1.96	2.03	2.48	2.90	2.21	2.08	2.40	4.72	4.94	4.53	3.61	3.86	4.38	3.48	3.27
	12 months (september 2023)															
	0.10	0.12	0.08	0.15	0.11	0.09	0.11	0.06	-	-	-	-	-	-	-	-
(-)-Catechin	3 months (december 2022)															
	15.37	14.16	14.08	12.14	9.41	12.81	15.27	16.47	6.52	10.54	15.25	16.42	12.84	16.38	15.37	14.74
	6 months (march2023)															
	10.91	10.03	8.59	8.25	5.26	7.30	7.18	8.42	3.02	6.15	8.93	7.72	4.35	5.62	6.57	4.28
	9 months (june 2023)															
	12.48	20.04	19.09	17.23	12.53	15.66	15.77	21.76	11.64	16.02	21.77	21.80	15.76	19.11	18.67	16.52
	12 months (september 2023)															
	5.62	8.58	8.97	7.84	5.98	6.00	6.17	5.42	1.78	3.09	8.99	16.77	13.20	11.83	11.62	8.69

(+)-Catechin	3 months (december 2022)															
	31.18	25.30	26.48	31.92	33.54	39.18	32.59	32.29	62.01	75.69	60.25	63.34	54.91	56.92	56.80	43.88
	6 months (march2023)															
	34.02	25.72	24.97	30.04	29.89	35.08	32.78	27.80	46.75	50.37	44.61	42.49	30.96	37.05	33.23	24.83
	9 months (june 2023)															
	58.90	60.54	63.20	73.78	73.12	86.66	77.87	81.82	126.79	123.35	112.95	117.62	85.77	97.99	84.36	56.26
	12 months (september 2023)															
	43.60	57.38	44.81	60.81	32.72	29.14	26.22	20.16	75.14	94.25	135.48	177.89	127.49	162.28	128.34	77.38
(-)-Epicatechin	3 months (december 2022)															
	40.27	32.65	33.30	43.14	28.05	44.00	36.09	37.64	39.95	47.80	44.61	52.19	43.23	45.44	40.56	29.39
	6 months (march2023)															
	31.16	24.73	23.66	30.37	19.03	30.36	26.76	23.34	27.99	33.08	32.13	32.58	19.42	24.18	23.03	12.35
	9 months (june 2023)															
	50.32	56.62	53.62	69.28	46.22	78.53	59.14	64.70	69.08	77.52	75.57	84.36	51.46	63.59	55.82	35.17
	12 months (september 2023)															
	18.05	20.69	15.30	23.66	9.78	12.44	11.24	8.44	15.73	19.40	30.30	22.90	28.46	42.82	31.40	17.35
Resveratrol	3 months (december 2022)															
	12.85	12.61	14.98	19.28	9.57	7.28	12.06	14.03	11.07	8.07	19.28	21.22	9.29	13.20	7.91	11.82
	6 months (march2023)															
	9.33	9.12	9.73	11.5	5.54	3.45	14.87	19.85	11.53	13.29	23.07	26.11	10.52	15.73	11.20	11.86
	9 months (june 2023)															
	7.58	9.15	7.33	10.33	9.94	7.30	15.33	12.46	11.20	13.20	14.23	11.77	13.68	19.13	12.37	12.13
	12 months (september 2023)															
	7.98	10.70	8.61	14.81	13.08	9.80	11.83	11.29	11.83	12.93	12.90	12.34	13.90	21.40	9.41	10.19
Ellagic acid	3 months (december 2022)															
	14.40	13.13	14.22	16.38	14.07	13.17	11.35	15.40	10.97	9.10	9.11	9.14	8.53	9.33	7.78	8.31
	6 months (march2023)															
	16.12	13.88	14.01	16.63	12.64	12.79	12.49	14.80	6.41	5.90	5.44	4.19	3.74	4.06	2.98	3.68
	9 months (june 2023)															
	34.85	37.76	38.55	46.41	41.83	42.68	35.97	53.82	29.78	24.68	23.07	22.06	19.00	20.45	14.93	14.45
	12 months (september 2023)															
	23.16	24.38	22.16	26.09	13.58	9.44	9.78	8.84	8.84	9.13	13.61	19.06	16.05	20.15	12.48	4.69

The anthocyanidins and anthocyanins values obtained by the HPLC-MS method from red wines
µg mL^{-1}

Compunds	FNI M	FNI V1	FNI V2	FNI V3	FNII M	FNII V1	FNII V2	FNII V3	CSI M	CSI V1	CSI V2	CSI V3	CSII M	CSII V1	CSII V2	CSII V3
Delphinidin	3 months (december 2022)															
	1.27	1.28	1.29	1.30	1.26	1.27	1.26	1.27	1.31	1.32	1.28	1.27	1.30	1.34	1.31	1.33
	6 months (march2023)															
	2.59	2.53	2.46	2.58	2.29	1.79	1.92	2.05	2.23	2.40	2.19	1.75	1.61	1.81	1.71	1.71
	9 months (june 2023)															
	2.13	2.18	2.17	2.23	2.09	1.24	1.83	1.81	2.99	2.98	2.28	2.44	2.69	3.83	3.00	3.73
	12 months (september 2023)															
	1.04	1.18	1.44	1.71	1.79	1.18	1.15	1.24	5.44	5.28	3.99	2.78	2.26	2.68	2.23	2.51
Delphinidin-3-glucoside	3 months (december 2022)															
	564.31	534.26	558.05	688.37	561.53	456.51	459.10	635.21	787.14	921.56	823.60	628.11	804.27	950.80	877.57	832.50
	6 months (march2023)															
	610.25	596.23	612.44	747.76	560.71	366.72	496.00	572.78	362.48	396.86	378.37	255.32	271.56	319.08	271.66	221.65
	9 months (june 2023)															
	282.78	261.25	244.87	289.96	265.47	138.53	200.35	206.87	205.69	248.11	179.08	125.73	265.05	380.24	331.31	303.83
	12 months (september 2023)															
	65.14	71.66	79.83	118.14	125.12	90.48	98.14	102.06	81.38	112.47	97.38	70.19	79.54	95.38	84.93	81.88
Peonidin-3-glucoside	3 months (december 2022)															
	25.23	24.66	27.24	31.24	25.41	27.05	23.30	22.28	22.09	26.03	24.70	20.72	25.41	29.10	26.50	26.88
	6 months (march2023)															
	20.80	20.15	20.29	26.04	18.40	22.45	19.07	21.79	12.89	8.34	14.64	10.35	11.01	12.50	11.57	10.05
	9 months (june 2023)															
	9.93	9.30	8.77	10.41	8.93	6.03	8.30	7.39	3.91	6.36	3.89	2.80	6.51	9.94	8.98	7.71
	12 months (september 2023)															
	5.46	6.27	7.96	11.80	12.32	1.15	11.54	10.63	6.14	9.93	8.92	7.19	7.39	9.21	7.90	8.05
Cyanidin-3-glucoside	3 months (december 2022)															
	1.80	2.07	2.42	3.19	2.32	2.18	1.85	2.70	4.32	6.39	5.78	3.82	5.21	7.30	6.25	6.03
	6 months (march2023)															
	2.93	3.06	3.28	4.24	2.88	2.49	2.68	3.76	2.77	4.27	3.84	2.30	2.51	3.38	2.71	2.77
	9 months (june 2023)															
	0.91	0.84	0.75	1.04	0.84	0.54	0.66	0.70	1.00	1.49	1.08	0.72	1.60	2.73	2.22	2.15
	12 luni (septembrie 2023)															
	0.97	1.18	1.57	2.08	2.06	1.78	1.78	1.94	1.58	2.46	2.07	1.32	1.44	2.14	1.81	1.91
Malvidin	3 months (december 2022)															
	4.27	4.13	4.28	4.38	3.93	3.38	3.10	3.53	17.19	18.97	19.72	19.76	23.93	22.71	20.13	18.92

	6 months (march2023)															
	2.85	2.92	2.67	3.04	2.77	2.04	2.30	2.61	4.77	6.86	6.16	5.70	4.96	9.89	10.07	6.07
	9 months (june 2023)															
	1.63	1.71	1.65	1.85	1.67	0.95	1.42	1.40	4.29	5.08	4.00	3.50	6.29	7.72	7.05	6.03
	12 months (september 2023)															
	2.24	1.94	1.86	2.80	2.39	1.85	2.07	1.88	5.04	6.66	6.21	5.91	5.88	5.54	4.88	4.03
Malvidin-3-glucoside	3 months (december 2022)															
	708.74	656.63	680.08	708.34	704.28	585.03	571.34	644.04	539.50	566.18	569.14	565.22	668.01	688.32	632.10	598.86
	6 months (march2023)															
	677.73	633.92	620.96	683.06	581.64	520.02	518.03	594.92	279.53	286.25	298.71	252.31	241.92	240.13	228.98	169.92
	9 months (june 2023)															
	357.36	374.58	353.70	407.61	371.60	199.06	306.44	306.81	166.71	214.19	157.23	139.34	246.61	288.05	264.10	229.34
	12 months (september 2023)															
	153.50	163.51	181.95	224.48	264.63	193.84	211.61	213.58	112.79	154.73	150.03	144.05	147.20	157.49	142.38	132.96
Petunidin-3-glucoside	3 months (december 2022)															
	92.75	87.00	91.11	104.66	92.46	78.86	78.16	92.82	93.84	101.07	91.60	79.19	93.91	106.97	98.08	97.23
	6 months (march2023)															
	79.92	77.86	78.04	94.34	73.93	63.93	68.14	75.85	34.72	35.33	34.13	25.70	25.81	28.37	25.05	22.97
	9 months (june 2023)															
	39.09	38.16	35.67	43.34	38.97	20.57	30.30	30.21	19.53	23.05	16.00	11.82	22.46	29.96	26.35	25.58
	12 months (september 2023)															
	36.23	37.38	44.72	63.72	71.73	52.13	56.35	56.03	31.49	42.66	17.17	29.17	30.36	36.71	32.12	33.18

FN- Feteasca Neagra, CS- Cabernet Sauvignon, I-punch down, II-pumping over, M-control samples, V1- 50, V2-100, V3-500 µg of melatonin were applied to 1 kg of must.
